# The challenge of COVID-19 and hematopoietic cell transplantation; EBMT recommendations for management of hematopoietic cell transplant recipients, their donors, and patients undergoing CAR T-cell therapy

**DOI:** 10.1038/s41409-020-0919-0

**Published:** 2020-05-13

**Authors:** Per Ljungman, Malgorzata Mikulska, Rafael de la Camara, Grzegorz W. Basak, Christian Chabannon, Selim Corbacioglu, Rafael Duarte, Harry Dolstra, Arjan C. Lankester, Mohamad Mohty, Silvia Montoto, John Murray, Régis Peffault de Latour, John A. Snowden, Ibrahim Yakoub-Agha, Bregje Verhoeven, Nicolaus Kröger, Jan Styczynski, for the European Society for Blood and Marrow Transplantation

**Affiliations:** 1 Department of Cellular Therapy and Allogeneic Stem Cell Transplantation, Karolinska University Hospital Huddinge, Stockholm, Sweden; 2 Division of Hematology, Department of Medicine Huddinge, Karolinska Institutet, Stockholm, Sweden; 3 Division of Infectious Diseases, University of Genoa and Ospedale Policlinico San Martino, Genova, Italy; 4 Department of Hematology, Hospital de la Princesa, Madrid, Spain; 5 Department of Hematology, Oncology and Internal Medicine, Medical University of Warsaw, Warsaw, Poland; 6 Institut Paoli Calmettes & Inserm CBT-1409, Centres d’Investigations Cliniques en Biothérapies, Marseille, France; 7 Pediatric Hematology, Oncology and Stem Cell Transplantation Department, University of Regensburg, Regensburg, Germany; 8 Servicio de Hematologia y Hemoterapia, Hospital Universitario Puerta de Hierro, Madrid, Spain; 9 Laboratory of Hematology, Department of Laboratory Medicine, Radboud University Medical Center, Nijmegen, the Netherlands; 10 Willem-Alexander Children’s Hospital, Department of Pediatrics, Leiden University Medical Centre Leiden, Leiden, the Netherlands; 11 Department of Hematology, Hospital Saint Antoine, Paris, France; 12 St. Bartholomew’s Hospital, Barts Health NHS Trust, London, UK; 13 The Christie NHS Foundation Trust, Manchester, UK; 14 BMT Unit, Department of Hematology, Hospital St. Louis, Paris, France; 15 Department of Haematology, Sheffield Teaching Hospitals NHS Foundation Trust, Sheffield, UK; 16 CHU de Lille, Univ Lille, INSERM, U1285 Lille, France; 17 Foundation Hematon, Utrecht, the Netherlands; 18 Department of Stem cell Transplantation, University Hospital Eppendorf, Hamburg, Germany; 19 Pediatric Hematology and Oncology, University Hospital, Collegium Medicum, Nicolaus Copernicus University Torun, Bydgoszcz, Poland

**Keywords:** Health care, Diseases

## Abstract

The new coronavirus SARS-CoV-2 has rapidly spread over the world causing the disease by WHO called COVID-19. This pandemic poses unprecedented stress on the health care system including programs performing allogeneic and autologous hematopoietic cell transplantation (HCT) and cellular therapy such as with CAR T cells. Risk factors for severe disease include age and predisposing conditions such as cancer. The true impact on stem cell transplant and CAR T-cell recipients in unknown. The European Society for Blood and Marrow Transplantation (EBMT) has therefore developed recommendations for transplant programs and physicians caring for these patients. These guidelines were developed by experts from the Infectious Diseases Working Party and have been endorsed by EBMT’s scientific council and board. This work intends to provide guidelines for transplant centers, management of transplant candidates and recipients, and donor issues until the COVID-19 pandemic has passed.

## Introduction

A novel coronavirus named SARS-CoV-2 of a zoonotic origin emerged in the beginning of this year and the disease called Coronavirus Disease 2019 (COVID-19) started spreading worldwide. The outbreak started in Wuhan in China and was initially mainly concentrated in Asia with a few import cases to other parts of the world. Next, a large number of cases started to appear in northern Italy. Thereafter, the outbreak has spread to Iran, all European countries, to North America, and the rest of the world. The WHO classified COVID-19 a pandemic on March 11. The pressure on health care systems is very high in all countries affected and increasing numbers of infected health care staff are being reported. Many European countries have imposed major restrictions on meetings, travel, and everyday life. This is likely to impact greatly on the HCT and CAR T activity in Europe as in many other parts of the world throughout 2020, and potentially beyond.

Early in the outbreak, the Infectious Diseases Working Party (IDWP) of the European Society for Blood and Marrow Transplantation (EBMT) started working on guidelines to support transplant centers in developing strategies for management based on existing experience. This work was performed in collaboration with the infectious diseases group of American Society of Transplantation and Cellular Therapy. In addition, the IDWP started collecting patient reports through the mechanism of the EBMT registry to rapidly collect information about outcome of autologous and allogeneic HCT patients developing COVID-19. The EBMT has also started educational activities directed to physicians, patients, and care givers through webinars. Five weekly updates of the recommendations have now been distributed and the current status is summarized in this paper.

## COVID-19

The infection has spread very rapidly in the population of several countries. The time from exposure to symptom development is between 2 and 14 days (median 5 days). Symptoms vary from no or very mild symptoms of an upper respiratory infection to very severe resulting in the need for intensive care and death from Acute Respiratory Distress Syndrome (ARDS). It is becoming increasingly clear that asymptomatic or very mildly symptomatic individuals are important for the rapid spread of the infection in the population. The risks both for infections and for severe disease seem to be lower in children. Increasing age and the presence of comorbidities, such as hypertension, cardiovascular disease, diabetes, and pulmonary disease, are reported risk factors for severe disease and mortality [1–6]. Patients, who develop more severe symptoms including respiratory failure, often progress during the 2nd week after the start of symptoms and it is believed that this is to a great extent due to an immune reaction in the lower airways. Whether patients, who are immunosuppressed develop a different form of disease is currently unclear although some preliminary information from early cases indicate that such can be the case. Health care workers are also at risk for contracting COVID-19 [7].

## Prevention policies and procedures

Since the COVID-19 situation varies substantially between and within countries, we recognize that centers are mandated to follow guidelines, policies, and procedures decided by national authorities as well as local and institutional policies. Avoiding exposure by adhering to recommended hygiene procedures, isolation of SARS-CoV-2-infected individuals, and social distancing especially for risk groups are currently the main prevention strategies utilized in most European countries. Limiting exposure of health care personnel and mitigating the psychological consequences of altered and stressful working conditions is another high priority to ensure that appropriate capacities remain available to treat patients in the middle term to long term.

We believe that patients having undergone HCT or are receiving CAR T-cell therapy can require specific considerations and we therefore outline some general principles of guidance, policies, and procedures having common themes, including but not limited to the following.

### Staff

Staff with any symptoms of infection should stay at home. Testing for SARS-CoV-2 is strongly recommended since symptoms can be uncharacteristic and very mild. Return to work by staff, who have recovered from COVID-19 should follow national guidelines, usually requiring the resolution of symptoms and two negative PCR results.

Training of staff in proper procedures, including caring for those with suspected or confirmed infection, ensuring adequate access to personal protection equipment and planning for possible staff shortage are critical. Personal protective equipment especially masks are important to limit the spread and to reduce the risk for health care workers to become infected. Surgical masks protect mainly for transmission of the virus from an infected individual while certain masks of the FFP2/3 class (those with an exhalation valve) protect the wearer of the mask but may not prevent from transmitting the virus. An FFP2/3 mask without exhalation valve also prevents from transmitting and is an alternative. Thus, correct selection of the mask and correct use are crucial. Many staff will be working extended hours in highly stressful circumstances and the emotional and psychological impact should not be underestimated. Regular team briefings and opportunities to talk to prevent carer fatigue should be in place.

### Outpatient visits and visitors

Outpatient visits should be as much as possible either deferred or substituted with telemedicine visits if deemed appropriate and feasible. Staff should preferably be dedicated to a COVID-19 free transplant unit and not used interchangeably to care for COVID-19 positive patients although in centers with a large number of COVID-19 positive patients, it is recognized that this will not be feasible. It is critical that proper protective equipment is used as recommended by national and international competent authorities.

No visitors should be allowed in transplant units. There might be exceptions for parents to transplanted children; testing for SAR-CoV-2 should then be considered before entering the ward. Repeated testing might then be necessary. This will bring its own set of challenges when attempting to have end of life conversations with families who will not be present in person. New ways of working and communicating with patients and families need to be identified, such as a daily telephone round of the next of kin to update on progress and transmit the patient’s wishes.

### Patients after HCT

Considerations in HCT and CAR T-cell recipients still being regarded as immunosuppressed or having significant organ dysfunction. They should limit their risk of exposure to infected individuals as much as possible and strictly adhere to prevention practices such as hand hygiene and social distancing. Stem cell transplant patients should refrain from travel and if travel is deemed absolutely necessary, travel by private car instead of any public transportations system including train, bus, or plane is recommended if feasible.

Physical and social isolation although a usual practice for many transplant patients will now extend further and for a longer period of time and local services and practices need to be explored by the nursing staff to ensure that patients have adequate provision to be cared for at home.

All patients, including those without symptoms, should be triaged and tested before entering the transplant ward. Adequate space for symptomatic patients while awaiting the results of COVID-19 testing should be allocated preferably separate from the transplant unit.

Patients planned to be admitted for a transplant or to undergo CAR T-cell therapy should try to minimize the risk by home isolation 14 days before the start of the transplant conditioning. Unnecessary clinic visits should be avoided.

### Transplant candidates

It is recognized that patients might suffer harm if necessary transplant and other treatment procedures are delayed due to COVID-19. It is not possible to give clear guidelines regarding which procedures should be delayed since the situation is highly variable between transplant centers. Before starting the transplant procedure, availability of adequately trained staff, ICU beds, ventilators, as well as availability of the stem cell product should be ensured. In general, however, non-urgent transplants should be deferred as much as possible, especially for non-malignant disorders. For those that will have delays in treatment, signposting to appropriate local advice and support groups is of paramount importance.

All patients should be tested for SARS-CoV-2 and the test results should be negative before start of the conditioning regardless of whether upper respiratory symptoms are present.

A difficult question based on lack of data is deferral of transplant candidates if they become infected with COVID-19. At the point of consent for transplant discussions around advanced care planning should be initiated and include the scenario of becoming positive during the transplant process and what steps and actions the patient and treating team wish to take, with early conversations and plans put in place as soon as there is a suspicion that the patient may be COVID-19 positive. The decision has to be made based on individual considerations taking into account the risk of the patient associated with on one hand the delay of the procedure and on the other proceeding with conditioning. In general, however, if a transplant candidate is diagnosed with COVID-19 a deferral of at least 3 months is advisable, in accordance with ECDC recommendations. However, this is not always possible due to the risk for progression of the underlying disease. This might be particularly pertinent for patients waiting for CAR T-cell therapy since this is frequently performed on patients refractory for other therapies and therefore being at a very high risk for progress of the underlying disease Therefore, in patients with high risk disease, stem cell transplantation should be deferred until the patient is asymptomatic and has two negative virus PCR swabs taken at least 24 h apart. Deferral of 14 days is a minimum but should preferably be 21 days and a new PCR is recommended before the start of conditioning. In patients with low-risk disease a 3-month HCT deferral is recommended. Individual considerations have to be made for CAR T-cell patients.

In case of close contact with a person diagnosed with COVID-19 any transplant procedures (PBSC mobilization, BM harvest, and conditioning) shall not be performed within at least 14, and preferably 21, days from the last contact. Patient should be closely monitored for the presence of COVID-19, with confirmed PCR negativity before any transplant procedure is undertaken.

### Donor considerations

Access to a stem cell donor might be restricted either due to the donor becoming infected, logistical reasons at the harvest centers in the middle of a strained health care system, or travel restrictions across international borders. It is therefore strongly recommended to have secured stem cell product access by freezing the product before start of conditioning, and, in situations when this is not possible, to have an alternative donor as a back-up. Peripheral blood should be preferentially used unless there is a strong indication for bone marrow.

SARS-CoV and MERS-CoV have been detected in blood, although there have not been any reports of transmission from donor to recipient either in transfusion of blood products or cellular therapies [8]. WMDA has produced recommendations and the EBMT endorses these guidelines.

In case of diagnosis of COVID-19, donor must be excluded from donation. At this time, it is not possible to give recommendations when such an individual can be cleared for donation, but 3-month deferral can be considered unless the need for donation is urgent, and case-specific considerations should be made.

In case of close contact with a person diagnosed with SARS-CoV-2, the donor shall be excluded from donation for at least 28 days. Donor should be closely monitored for the presence of COVID-19. If the patient’s need for transplant is urgent, the donor is completely well, a test is negative for SARS-CoV-2 and there are no suitable alternative donors, earlier collection may be considered subject to careful risk assessment.

Donors within 28 days of donation should practice good hygiene and be as socially isolated as feasible during this period. Unnecessary travel should be avoided. Donors should have been asymptomatic for at least 14 (preferably 21) days before donation. It is recommended that donors are tested for COVID-19 prior to starting the mobilization procedure.

## Diagnosis and management of COVID-19

### Diagnosis of COVID-19

Diagnostic procedures for COVID-19 should follow national or local guidelines. It is important to note that a test for SARS-CoV-2 in nasopharyngeal swab can be falsely negative and needs to be repeated if there is a strong clinical suspicion of COVID-19. The performance of testing is better in samples from the lower than from the upper respiratory tract. It is also important to test for other respiratory viral pathogens including influenza and RSV preferably by multiplex PCR.

### SARS-CoV-2 infected patients

Patients, who are positive for SARS-CoV-2 should not be treated in rooms with laminar air flow or other rooms (HEPA) with positive pressure unless the ventilation can be turned off. All patients positive for SARS-CoV-2 in an upper respiratory tract sample should undergo chest imaging, preferably by CT, and evaluation of oxygenation impairment. Routine bronchoalveolar lavage is not recommended if a patient tested positive for SARS-CoV-2 given risk of transmission amongst health care workers, unless a co-infection is suspected. Co-pathogens should be evaluated and treated.

### Treatment

#### Treatment of COVID-19 positive transplant recipients and CAR T-cell patients

Currently there is no approved treatment options in Europe and there is no available vaccine. Several drugs have been studied in prior coronavirus outbreaks (SARS-CoV and MERS-CoV) and though some benefit has been demonstrated, the data are inconclusive. Remdesivir has demonstrated in vitro and in vivo activity in animal models against the viral pathogens MERS and SARS, which are also coronaviruses and are structurally similar to SARS-CoV-2. The limited preclinical data on remdesivir in MERS and SARS indicate that remdesivir may have potential activity against COVID-19 [9–11]. Remdesivir is now approved by the FDA, and the EMA has recently granted compassionate use. Lopinavir/ritonavir has also been used but a recently published trial failed its primary endpoint [10, 12]. Chloroquine and hydroxychloroquine have also been used with early data suggesting reduction of viral load and have been used for therapy [9, 13, 14]. Recently, several competent authorities have expressed concerns about safety and studies have reported opposing results. Interferon-α and -β are also being studied.

Since an important part of the pathology seems to be cytokine release, different therapies addressing this syndrome have been tested. Tocilizumab, which is approved for cytokine release syndrome after CAR T-cell therapy, have been used and is approved in China [15, 16]. Data on the use of corticosteroids are contradictory. However, short-term corticosteroid therapy was associated with lower mortality in immunocompetent patients with COVID-19-associated ARDS.

Some data suggest that use of angiotensin conversion enzyme inhibitors and angiotensin II receptor blocker might be implicated in development of organ failure in COVID-19 patients [17, 18]  but lower mortality has also been reported with the use of these drugs [19]. Similarly, it has been suggested that NSAIDs might have negative effects and therefore acetaminophen/paracetamol are preferred as anti-pyretics.

At this point no clear recommendations can be made on specific therapies due to limited data and unknown risk vs benefit. Even less data are available for pediatric patients. Therapy should be given in close collaboration with specialists in infectious diseases.  Despite FDA approval of remdesivir, it is still unclear if and when it optimally should be used. Later in the course of the infection anti-inflammatory therapy with corticosteroids and/or tocilizumab has been shown to be of value in non-transplant patients. Supportive care is crucial including non-invasive ventilation and anti-coagulants to prevent thromboembolic complications, which can be frequent and severe in patients with COVID. Treatment of viral, bacterial and fungal co-pathogens should be optimized. It is currently recommended that immunosuppressive prophylaxis/treatment is continued since there is no data supporting reducing immunosuppression and it might instead cause harm.

## JACIE and COVID-19

In the light of substantial and ongoing disruption caused by the international COVID-19 pandemic, which has made normal JACIE accreditation services difficult or impossible to maintain in the foreseeable future, the EBMT took the decision to extend current accreditations by 12 months and to give coverage to those centers that are actively preparing for reaccreditation https://www.ebmt.org/covid-19-and-bmt.

Even so, for centers already accredited (or preparing for accreditation) current and recent versions of JACIE standards include extensive reference to disaster planning within the clinical, collection (apheresis and bone marrow) and processing sections [20]. Modifications to usual care should be considered within the broader organization according to ongoing quality management systems in the program and other JACIE accreditation requirements. JACIE is currently evaluating whether more simplified versions of quality standards currently being applied to low and middle income countries, such as ‘Stepwise’ or other systems, will have value during the COVID-19 pandemic and its aftermath which may last for many months.

## Discussion

The COVID-19 pandemic has resulted in previously unprecedented challenges to transplant programs and physicians performing stem cell transplantation. Although WHO and other international and national authorities have warned for the possibility of a new pandemic caused most likely by a viral pathogen such as SARS-CoV-1, SARS-CoV-2, a new influenza strain, or possibly a hemorrhagic fever such as Ebola or Lassa, the health care systems were caught unprepared for the COVID-19 pandemic. However, enormous activity has started from the beginning of the outbreak in China to learn more about the virus and the disease and to disseminate this knowledge across the world, Since the virus is new to the human population, it is likely that before long-term virus control is achieved a large proportion of the population will have to either have experienced the viral infection or have been vaccinated. Therefore, continued international collaboration is necessary to increase our knowledge and thereby decrease the risk for harm to the very vulnerable group of patients either waiting for or having undergone the potentially life-saving procedure of HCT or cellular therapy. Scientific organizations such as the EBMT has a responsibility to the community to help gather and disseminate information including collecting cases of COVID-19 through its well established registry and also to actively participate in educational activities directed to all stakeholders in the transplant and cellular therapy activities.

On a national level, EBMT cannot define the specifics of how centers need to work internally and with each other within networks and public health and the broader context of health care. Some of aspects of networking and working with public health and other stakeholders may not come easy or be straightforward. However, irrespective of center, nation or health service, transplant teams should work to put patients’ interests first, values which are central to the Mission and Vision of EBMT.

Finally, we recommend that you follow https://www.ebmt.org/covid-19-and-bmt and participate in the EBMT IDWP prospective survey of the impact of COVID-19 on patients undergoing HCT and cell therapy.
